# Supplementary material for: Identification of photoperiod-induced specific miRNAs in the adrenal glands of Sunite sheep (Ovis aries)
Source: Front Vet Sci. 2022 Jul 22;9:888207. doi: 10.3389/fvets.2022.888207 (PMC9354845; doi:10.3389/fvets.2022.888207)
Supplement: Supplementary file 1 [file Data_Sheet_1.ZIP › Supplementary file/miRDeep2 file.zip!/miRDeep2 file/Know_Structure/SPLP42A3/oar-mir-143.pdf]

miRBase precursor : oar-mir-143
Total read count : 302409
oar-miR-143 read count : 301237
remaining reads : 1172

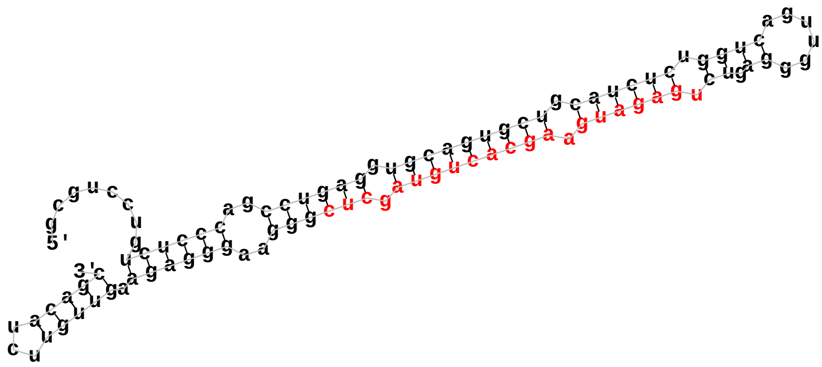

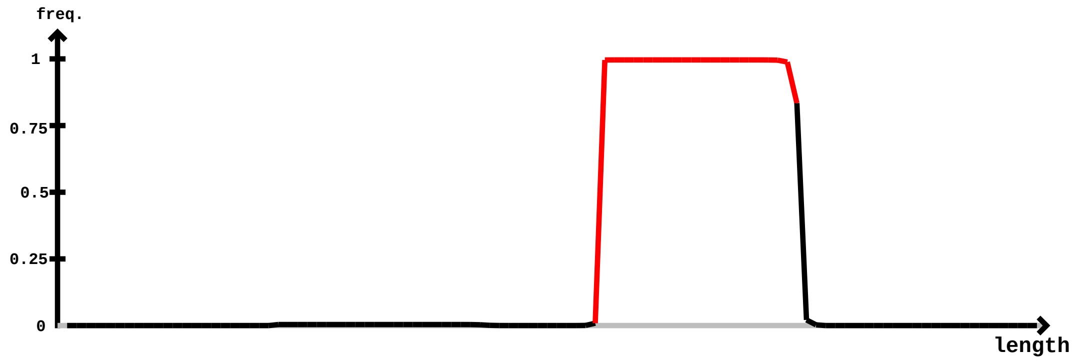

oar-miR-143

```
5'- gcguccugucucccagccugaggugcagugcugcaucucuggucaguugggagucugagaugaagcacuguagcucgggaagggagaaguuguucuacagc  -3'    exp
    ........((((((..((((((.(((((((((.((((((.((((......)).)).)))))).))))))))).))))))..)))))).(((((...)))))   reads   mm   sample
    .....................ggugcagugcugcaucucu.............................................................   4       0    seq
    .....................ggugcagugcugcaucucug............................................................   4       0    seq
    .....................ggugcagugcugcaucucugg...........................................................   241     0    seq
    .....................ggugcagugcugcaucucuggu..........................................................   641     0    seq
    .....................ggugcagugcugcaucucugguc.........................................................   263     0    seq
    .....................ggugcagugcugcaucucuggucа........................................................   13      0    seq
    .....................ggugcagugcugcaucucuggucagu......................................................   1       0    seq
    ......................gugcagugcugcaucucugguc.........................................................   2       0    seq
    .......................ugcagugcugcaucucuggucagu......................................................   1       0    seq
    ..........................................ucaguugggagucugagaugaagcacuguagcuc.........................   1       0    seq
    .....................................................ucugagaugaagcacuguagc...........................   3       0    seq
    .....................................................ucugagaugaagcacuguagcu..........................   7       0    seq
    .....................................................ucugagaugaagcacuguagcuc.........................   126     0    seq
    ......................................................cugagaugaagcacugu..............................   4       0    seq
    ......................................................cugagaugaagcacugua.............................   1       0    seq
    ......................................................cugagaugaagcacuguag............................   4       0    seq
    ......................................................cugagaugaagcacuguagc...........................   30      0    seq
    ......................................................cugagaugaagcacuguagcu..........................   451     0    seq
    ......................................................cugagaugaagcacuguagcuc.........................   2059    0    seq
    ......................................................cugagaugaagcacuguagcucg........................   12      0    seq
    .......................................................ugagaugaagcacugua.............................   59      0    seq
    .......................................................ugagaugaagcacuUua.............................   1       1    seq
    .......................................................ugagaugaagUacugua.............................   1       1    seq
    .......................................................ugagaugaagcacuguG.............................   1       1    seq
    .......................................................ugagaugGagcacugua.............................   2       1    seq
    .......................................................ugagaugaagcacuguag............................   195     0    seq
    .......................................................ugagaugaagcacuguagc...........................   2126    0    seq
    .......................................................ugagaugaagcacuguagcu..........................   46159   0    seq
    .......................................................ugagaugaagcacuguagcuc.........................   243542  0    seq
    .......................................................ugagaugaagcacuguagcucg........................   5567    0    seq
    .......................................................ugagaugaagcacuguagcucgg.......................   798     0    seq
    .......................................................ugagaugaagcacuguagcucggg......................   3       0    seq
    .......................................................ugagaugaagcacuguagcucggga.....................   1       0    seq
    ........................................................gagaugaagcacuguagc...........................   1       0    seq
    ........................................................gagaugaagcacuguagcu..........................   8       0    seq
```

oar-miR-143

```
gcguccugucucccagccugaggugcagugcugcaucucuggucaguugggagucugagaugaagcacuguagcucgggaagggagaaguuguucuacagc
........................................................gagaugaagcacuguagcuc.........................      59      0      seq
........................................................gagaugaagcacuguagcucg........................      1       0      seq
.........................................................agaugaagcacuguagcu..........................      2       0      seq
.........................................................agaugaagcacuguagcucgg.......................      11      0      seq
..........................................................gaugaagcacuguagcucgg.......................      1       0      seq
..........................................................gaugaagcacuguagcucggga.....................      2       0      seq
............................................................ugaagcacuguagcucgggaag...................      1       0      seq
```
